# Effect of CYP3A5*3, ABCC2 C-24T, and ABCC2 C3972T Genetic Polymorphisms on Direct Cost of Kidney Transplant Recipients

**DOI:** 10.7759/cureus.69221

**Published:** 2024-09-11

**Authors:** Chiau Ling Choong, Farida Islahudin, Mohd Makmor-Bakry, Nor Asyikin Mohd Tahir, Hin-Seng Wong, Rosnawati Yahya

**Affiliations:** 1 Centre of Quality Medicine Management, Faculty of Pharmacy, Universiti Kebangsaan Malaysia, Kuala Lumpur, MYS; 2 Department of Nephrology, Sunway Medical Centre, Petaling Jaya , MYS

**Keywords:** abcc2, cost-effectiveness, cyp3a5, kidney transplant recipients, polymorphism

## Abstract

Introduction

Genetic variations can influence how kidney transplant recipients (KTRs) respond to immunosuppressive drugs. However, limited resources necessitate a cost-benefit analysis of pharmacogenetic testing to determine its role in routine practice. This study investigated the cost-effectiveness of three genetic polymorphisms (CYP3A5*3, *ABCC2 -24C>T*, and* ABCC2 3972C>T*) in KTRs.

Methods

This was a multicenter, prospective observational cohort study that included patients on tacrolimus-mycophenolate-prednisolone treatment. Ethnically diverse adult KTRs who had undergone kidney transplantation between 2020 and 2021 and consented were enrolled in the study. Deoxyribonucleic acid (DNA) was extracted from the collected blood samples using a commercially available kit. CYP3A5*3, *ABCC2 -24C>T*, and *ABCC2 3972C>T* single nucleotide polymorphisms (SNPs) were determined by polymerase chain reaction (PCR).

Results

Data was analyzed from 39 KTRs with an average age of 32.2 ± 7.0 years. The median annual healthcare cost per patient was MYR 52,700 (laboratory tests and immunosuppressants being the highest expenses). Notably, the annual cost was significantly higher in patients with the CYP3A5*3 variant compared to the wildtype (p < 0.001). Furthermore, an incremental cost-effectiveness analysis revealed that carriers of the CYP3A5*1 wildtype allele, the *ABCC2 -24C>T T *variant allele, and the *ABCC2 3972C>T T* variant allele were associated with a more cost-effective approach to kidney transplantation management, potentially reducing the risk of graft rejection and acute tubular necrosis (ATN).

Conclusion

While these findings suggest potential cost benefits for specific genotypes, further research with larger and more diverse patient populations is necessary to definitively establish the role of pharmacogenetic testing in optimizing cost-effectiveness for KTRs.

## Introduction

Kidney transplantation is the preferred treatment for end-stage kidney disease [1]. It has proven to be able to provide better survival, aid in extending lifespan, and enhance quality of life, offering a compelling economic advantage compared to long-term dialysis [1]. It was reported in Finland in recent years that the total annual costs were euro (EUR) 53,275 (range EUR 26,019 to 119,913) per patient in dialysis, EUR 59,583 (range EUR 14,966 to 232,479) for the first post-transplantation year (p < 0.001), and EUR 12,045 (range EUR 1,787 to 231,112) for the subsequent transplantation years (p < 0.001) compared to dialysis [1]. In Malaysia, the average cost that includes pretransplant work-up, transplant operation, and costs for the first year of renal transplantation was Malaysian Ringgit (MYR) 83,735 (US Dollars (USD) 24,452) for living-related renal transplantation and MYR 144,4475 (USD 42,185) for deceased donor renal transplantation [2]. While kidney transplantation offers significant cost benefits compared to dialysis in the long term, it can still represent a significant financial burden for kidney transplant recipients (KTRs). The need for long-term immunosuppression treatment remains a financial burden for both the individual and the healthcare system. In view of the high treatment costs, many strategies have been used over the years to reduce the cost of kidney transplantation. Among them, some countries advocate for the use of less expensive generic immunosuppressive medications. To significantly reduce the overall cost of post-transplant care without compromising patient outcomes, individualizing drug dosages based on a patient’s unique genetic makeup can further optimize post-transplant therapy [3].

It is indeed crucial for KTRs to be maintained with immunosuppressants long term. Immunosuppressive medications are incredibly effective at preventing acute rejection, but their side effects and high complication rates can have detrimental long-term effects, and this can have a negative effect on KTRs [4]. It was demonstrated that delayed graft function (DGF) and infection post-transplant are the most frequent complications. DGF is commonly caused by acute tubular necrosis (ATN) during the early period after transplantation, which is related to ischemic injury to the transplanted kidney [5]. DGF may be potentiated when there is a combined presence of ATN, acute rejection, and drug toxicity, which are the common causes of graft dysfunction [5]. Other possible complications due to adverse reactions of immunosuppressants are diabetes, hypertension, infection, nephrotoxicity, and malignancy. Thus, there has been an increasing interest in kidney transplantation precision medicine in order to provide safe and effective personalized immunosuppression based on the individual’s genetic makeup to optimize the effectiveness of treatment [6].

Pharmacogenetic testing, such as *CYP3A5* genotyping, allows for the implementation of personalized medication regimens. This approach facilitates the identification of the optimal therapeutic dose for individual patients, thereby minimizing the need for subsequent dose adjustments to achieve target drug concentrations [7]. Consequently, this strategy not only reduces the incidence of medication errors and adverse drug reactions (ADRs) but also minimizes medication waste and the requirement for additional treatments to manage complications arising from ADRs. In a clinical investigation by Thervet et al., *CYP3A5* genotype-guided tacrolimus dosing resulted in a significantly higher proportion of patients achieving targeted therapeutic levels at an earlier time point (day 3 post-initiation), with a concomitant decrease in dose modifications and a lower frequency of ADRs [7]. However, interethnic variability must be considered. While *CYP3A5* genotyping may reduce ADR-related healthcare expenditures for some patient populations, a study by Khan et al. suggested a potential increase in allograft rejection risk among Asian individuals with specific *CYP3A5* genotypes [8]. Therefore, pharmacogenetic information represents a valuable tool for optimizing medication management strategies, potentially reducing healthcare costs and improving patient outcomes. However, its application should be undertaken in conjunction with a comprehensive evaluation of all relevant clinical factors.

Genetic polymorphisms in drug transporters such as ABCC2 also play a critical role in the metabolism and disposition of these drugs [9,10]. While the impact on clinical outcomes in kidney transplantation necessitates further investigation, recent studies are elucidating crucial associations. *ABCC2 3972C>T* polymorphism further exemplifies the impact of pharmacogenetics on patient outcomes. Thishya et al. demonstrated a protective association between the homozygous CC genotype and infection risk following mycophenolic acid (MPA) administration [10]. This suggests that the variant influences MPA disposition, potentially reducing its immunosuppressive effects and lowering infection susceptibility. Identifying patients with this genotype could enable clinicians to proactively manage infection risk in KTRs, potentially leading to improved patient outcomes and reduced healthcare resource utilization. Choong et al. demonstrated a statistically significant association between the wild-type *ABCC2 -24C>T CC* genotype and an increased risk of acute graft rejection and ATN in a Malaysian KTRs population, compared to individuals carrying variant alleles [11], although other work has shown that Caucasian KTRs showed no association in the number of acute rejections, despite variant carriers of *ABCC2 -24C>T* showing significantly higher dose-corrected MPA trough levels [12]. This groundbreaking work paves the way for more extensive research to confirm the long-term clinical impact of *ABCC2 -24C>T* in kidney transplantation outcomes. However, the routine implementation of pharmacogenetic testing remains limited, potentially due to the absence of robust evidence demonstrating its cost-effectiveness in KTR management [13]. A more comprehensive understanding of the true economic burden associated with kidney transplantation, including the potential cost savings attributable to personalized medicine approaches, could facilitate a more realistic application of these tests in the clinical setting. However, the cost-effectiveness of such a personalized medicine approach based solely on pharmacogenetics remains unclear and warrants further investigation.

When determining the cost-effectiveness of a treatment, there are tangible medical expenses directly related to the transplant procedure and post-operative care. This can be further broken down into both direct and indirect costs. Among direct costs are pre-transplant workup, including patient suitability, blood tests, imaging studies, and tissue typing; surgical costs, including clinical fees and anesthesia; immunosuppressive medication; hospital stay; and post-transplant care [14]. Indirect costs may include non-medical expenses that patients and their families may incur due to a kidney transplant. This also includes travel and accommodation, loss of work income, psychological support, dietary changes, and loss of productivity [14]. While the initial focus often falls on direct medical costs, indirect costs can significantly impact patients’ financial burden and overall well-being. Determining both aspects contributes to a more comprehensive picture of the costs associated with kidney transplantation.

Although various works have shown the benefits of pharmacogenetic screening, there is little evidence to show cost-effectiveness. As a result, it is critical to investigate the costs associated with genotype-guided personalized strategies in order to determine a cost-effective strategy for overall transplant care. Therefore, this work aims to identify the cost-effectiveness of CYP3A5*3, *ABCC2 -24C>T*, and *ABCC2 3972C>T* genetic polymorphisms among KTRs with the hope that the findings can assist in guiding resource allocation in KTRs management.

## Materials and methods

A multicenter, cross-sectional cohort study involving adult KTRs who had undergone kidney transplantation between the years 2020 and 2021 was conducted in two major kidney transplant centers in Malaysia: General Hospital, Kuala Lumpur, and Selayang Hospital, Selangor. Written informed consent was obtained from every subject prior to their participation in this study. Patients were recruited if they were taking tacrolimus-MPA immunosuppressive agents for at least 12 months following kidney transplantation with available genetic data as performed from previous work [11]. Patients with incomplete clinical and cost data were excluded.

The study protocol was approved by the Universiti Kebangsaan Malaysia Research Ethics Committee (UKM PPI/111/8/JEP-2022-431) and registered under the National Medical Research Register, Ministry of Health Malaysia (NMRR ID-22-00076-D3H (IIR)). This study was conducted in compliance with ethical principles outlined in the Declaration of Helsinki and the Malaysian Good Clinical Practice Guidelines. The study report follows the Strengthening the Reporting of Pharmacogenetic Studies guidelines [15].

Data collection

After obtaining written informed consent from subjects, demographic data, clinical information, and medication intake for one year after transplantation were collected from the hospital’s electronic medical record and chart review; the demographic data collected included age, gender, ethnicity, and body weight.

The clinical information was composed of medical records of pre-transplant variables, namely primary diagnosis, co-morbidities, blood pressure reading prior to transplant, dialysis modality, dialysis duration, and types of transplants. Patient kidney function, denoted as the estimated glomerular filtration rate, was calculated using the Chronic Kidney Disease Epidemiology Collaboration equation [16]. Body weight, blood pressure reading, tacrolimus daily dose and its corresponding tacrolimus trough level, and MPA doses were recorded. Other data included were the presence of DGF, defined as the need for dialysis within the first week after transplantation [17], acute rejection diagnosed as biopsy-proven acute rejection or clinical acute rejection, calcineurin inhibitor toxicity, chronic allograft nephropathy, post-transplant diabetes mellitus and hypertension, hospital admission due to infection, transaminitis, diarrhea, malignancy, cytomegalovirus infection, BK virus infection, urinary tract infection, ATN, and leukopenia.

Details of the medication regimen from transplant to one year after transplant were noted from medical records. For medications taken prior to transplant, only the total number of medications taken was recorded for each patient.

Deoxyribonucleic acid (DNA) extraction and single nucleotide polymorphism (SNP) genotyping

Following informed consent, 39 peripheral blood samples were taken from the study participants, and the genomic DNA was extracted using a Qiagen DNeasy blood and tissue extraction kit (Qiagen, Hilden, Germany) in compliance with the DNA extraction protocol recommended by the manufacturer. Next, the purity of the extracted genomic DNA was examined [18]. Polymerase chain reaction (PCR) was used for genotyping the *CYP3A5* polymorphism 12083A>G and the *ABCC2* polymorphisms -24C>T and 3972C>T. The PCR samples were separated based on size and recorded using an E-Gel® Safe ImagerTM Realtime Transilluminator (Life Technologies, Kiryat Shmona, Israel) [19]. Prior to DNA Sanger sequencing, the PCR product was purified using a commercially available PCR purification kit (Applied Biosystems, UK). Purified DNA fragments were analyzed using the BigDye® Terminator version 3.1 cycle sequencing kit, which was run on a 96-capillary 3730xl DNA Analyzer at First BASE Laboratories Sdn. Bhd., Seri Kembangan, Malaysia (developed by Applied Biosystem, USA, and produced by Thermo Fisher Scientific). The DNA sequences of the SNP results were transcribed using the Sequence Scanner version 2.0 software (Applied Biosystems) and compared to the reference sequences in the Basic Local Alignment Search Tool program to confirm the presence of the polymorphism [20]. Three randomly selected DNA samples were genotyped and sent for direct sequencing from the subjects’ pool. Sequencing results confirmed the validity of the method.

Direct costs 

Patients that had complete demographic, medical, and medication data were then interviewed to determine the direct costs during transplantation. Data on patients’ costs were collected using a standardized form, which was tested prior to the study to ensure applicability.

Data on the direct medical costs and the frequency encountered by each patient were collected [1,21]:

- Outpatient visits

- Diagnostic procedures (inclusive of laboratory services, any medical imaging or procedures)

- Renal biopsies

- Hospitalizations

- Prescribed medications, namely ISA and all other medications

In Malaysia, the government healthcare facilities are fully subsidized. As such, the actual expenses for patients may not reflect the actual medical expenses involved for cost estimation [22]. The one-year medical cost was estimated through prices of resources obtained from publicly available information or published literature [22] (Table 1).

**Table 1:** Source of direct cost data and method used for cost estimation Source: Attorney General’s Chambers Malaysia 2014

Item	Unit cost (MYR 2020)	Data source	Calculation method
Outpatient clinic visit	120	Attorney General’s Chambers Malaysia. Fees (Medical) (Cost of Services) Order 2014. Federal Government Gazette; 2014.	Number of clinic visits multiplied by cost per visit
Drugs	Various	Ministry of Health price list	Price per unit of drug multiplied by total doses
Laboratory tests and investigations	Various	Attorney General’s Chambers Malaysia. Fees (Medical) (Cost of Services) Order 2014. Federal Government Gazette; 2014.	Cost multiplied by the number of tests done
Hospitalization	260	Attorney General’s Chambers Malaysia. Fees (Medical) (Cost of Services) Order 2014. Federal Government Gazette; 2014.	Cost per day multiplied by number of days of admission
Renal biopsy	500	Attorney General’s Chambers Malaysia. Fees (Medical) (Cost of Services) Order 2014. Federal Government Gazette; 2014.	Cost multiplied by number of procedures done

Study endpoint

To determine the cost-effectiveness of the study, the outcome of interest was achieving graft survival with acute graft rejection and/or ATN. Graft survival was defined as (i) graft survival without acute graft rejection and/or ATN was survival of transplant without the return to long-term dialysis, graft nephrectomy, and re-transplantation and/or diagnosis of ATN [23], or (ii) graft survival with acute graft rejection and/or ATN was defined as graft survival with an incidence of acute graft rejection (diagnosed as biopsy-proven acute rejection or clinical acute rejection) and/or diagnosis of ATN but without the return to long-term dialysis, graft nephrectomy, and re-transplantation.

Data analyses

Cost-effectiveness was calculated via incremental cost-effectiveness ratio (ICER) utilizing these costs. The formula to calculate ICER is as follows:

ICER = (cost of comparator A − cost of comparator B)/(effects of comparator A − effects of comparator B)

The numerator is described in monetary units, while effects are measured in terms of health status or another outcome of interest.

The cost-effectiveness for each genetic polymorphism, which includes CYP3A5*3, *ABCC2 -24C>T*, and *ABCC2 3972C>T* for graft survival with acute graft rejection and/or ATN, was calculated based on ICER using the average values obtained from patients. The formula to calculate ICER is as follows [24]:

ICER (graft survival with acute graft rejection and/or ATN)

= [median annual healthcare cost of (CYP3A5*3 polymorphism − CYP3A5*1 polymorphism)]/[proportion achieved graft survival without acute graft rejection and/or ATN in (CYP3A5*3 polymorphism − CYP3A5*1 polymorphism)] 

= [median annual healthcare cost of (*ABCC2 -24C>T T* polymorphism − *ABCC2 -24C>T C* polymorphism)]/[proportion achieved graft survival without acute graft rejection and/or ATN in (*ABCC2 -24C>T T* polymorphism − *ABCC2 -24C>T C* polymorphism)] 

= [median annual healthcare cost of (*ABCC2 3972C>T T* polymorphism − *ABCC2 3972C>T C* polymorphism)]/[proportion achieved graft survival without acute graft rejection and/or ATN in (*ABCC2 3972C>T T* polymorphism − *ABCC2 3972C>T C* polymorphism)] 

The calculated ICERs were then illustrated in a cost-effectiveness plane, which includes a line with a slope that is equal to the maximum cost-effectiveness threshold in Malaysia of MYR 19,929 to MYR 28,470 [25]. This is an established Malaysian cost-effectiveness threshold determined that reflects the specific needs and the economic and disease burden of the general population in Malaysia [25]. The horizontal axis of the cost-effectiveness plane represents the difference in health effects, whereas the vertical axis represents the difference in costs [26].

For this study, the wildtype CYP3A5*1, *ABCC2 -24C>T C*, and *ABCC2 3972C>T C* genetic polymorphisms were placed at the origin of the cost-effectiveness planes. The location of the variant CYP3A5*3, *ABCC2 -24C>T T*, and *ABCC2 3972C>T T* plots were placed based on the magnitude of the difference in cost and the difference in health effects between the wildtype allele and the variant allele. The ICERS were inspected based on the location relative to the maximum cost-effectiveness threshold plotted in the cost-effectiveness planes. The variant CYP3A5*3, *ABCC2 -24C>T T*, and *ABCC2 3972C>T T* were considered cost-effective if the plots were located to the right of the maximum cost-effectiveness threshold line found on the plane and were considered to be not cost-effective if the plots were located to the left of the maximum cost-effectiveness threshold line [27].

## Results

Patient characteristics

A total of 39 KTRs were recruited in this study, of which 21 (53.8%) were males. The mean age of the study cohort was 32.2 ± 7.0 years. The majority were Malays (n = 25, 64.1%) and living-related kidney transplantation (n = 30, 76.9%). The majority (n = 35, 89.7%) received basiliximab as the inductive agent. Thirty (77.0%) KTRs had graft survival without acute graft rejection and/or ATN. Different characteristics of wildtype and variant alleles and genotypes of *CYP3A5*, *ABCC2 -24C>T*, and *ABCC2 3972C>T* (Table 2) were compared. There were no statistically significant differences between the characteristics of the alleles and genotypes across all studied genes. The distribution of graft survival with or without acute graft rejection and/or ATN, demographics, and clinical information by allele and genotype status are shown in Table 2.

**Table 2:** Demographic and clinical characteristics of participants by CYP3A5*3, ABCC2 -24C>T, and ABCC2 3972C>T polymorphism (n = 39)

Parameter	All patients (n = 39)	CYP3A5*1 allele (wildtype), (n = 28)	CYP3A5*3 allele (variant), (n = 50)	*ABCC2 -24C>T C *allele (wildtype), (n = 62)	*ABCC2 -24C>T T* allele (variant), (n = 16)	*ABCC2 3972C>T C* allele (wildtype), (n = 56)	*ABCC2 3972C>T T* allele (variant), (n = 22)
Demographics							
Age at transplant (years), mean±SD	32.2 ± 7.0	31.3 ± 6.3	32.7 ± 7.3	32.0 ± 7.1	32.9 ± 6.5	32.5 ± 7.2	31.6 ± 6.4
Gender, n (%)							
Male	21 (53.8)	14 (50)	28 (44.0)	35 (56.5)	7 (43.8)	30 (53.6)	12 (54.5)
Female	8 (20.5)	14 (50)	22 (56.0)	27 (43.5)	9 (56.2)	26 (46.4)	10 (45.5)
Ethnicities, n (%)							
Malay	25 (64.1)	19 (67.9)	31 (62.0)	38 (61.3)	12 (75.0)	34 (60.7)	16 (72.8)
Chinese	5 (12.8)	2 (7.1)	8 (16.0)	8 (12.9)	2 (12.5)	7 (12.5)	3 (13.6)
Indian	9 (23.1)	7 (25.0)	11 (22.0)	16 (25.8)	2 (12.5)	15 (26.8)	3 (13.6)
Others	0 (0.0)	0 (0.0)	0 (0.0)	0 (0.0)	0 (0.0)	0 (0.0)	0 (0.0)
Clinical							
Type of donor, n (%)							
Living-related	30 (76.9)	23 (82.1)	37 (74.0)	50 (80.6)	10 (62.5)	46 (82.1)	14 (63.6)
Cadaveric	9 (23.1)	5 (17.9)	13 (26.0)	12 (19.4)	6 (37.5)	10 (17.9)	8 (36.4)
Induction, n (%)							
Basiliximab	35 (89.7)	26 (92.9)	44 (88.0)	56 (90.3)	14 (87.5)	50 (89.3)	20 (90.9)
ATG	4 (10.3)	2 (7.1)	6 (12.0)	6 (9.7)	2 (12.5)	6 (10.7)	2 (9.1)
Graft survival with acute graft rejection/ATN, n (%)	9 (23.0)	6 (21.4)	12 (24.0)	16 (25.8)	2 (12.5)	13 (23.2)	5 (22.7)
Graft survival without acute graft rejection/ATN, n (%)	30 (77.0)	22 (78.6)	38 (76.0)	46 (74.2)	14 (87.5)	43 (76.8)	17 (77.3)

Direct Costs

Table 3 shows the overall healthcare cost incurred by the patients. The median annual cost incurred per patient was MYR 52,700.72 (IQR: 46,085.61-60,746.63). From here, it was observed that laboratory tests incurred the highest cost, with a median annual cost of MYR 21,349.00 (IQR: 21,349.00-21,249.00) per patient. This was followed by ISA prescribed, which recorded a median annual cost of MYR 19,432.66 (IQR: 14,366.70-26,339.83) per patient.

**Table 3:** Median annual direct costs for outpatient, laboratory tests, hospitalization, biopsies, ISA, and other medications of the study population (n = 39) Note: cost in the year 2021 ISA, immunosuppressive agents; IQR, interquartile range; MYR, Malaysian Ringgit

Cost component	MYR, median (IQR)
Outpatient visit	6,360.00 (6,000.00-6,360.00)
Laboratory tests	21,349.00 (21,349.00-21,429.00)
Hospitalization	4,344.00 (780.00-6,907.80)
Biopsies	500.00 (500.00-500.00)
ISA	19,432.66 (14,366.70-26,339.83)
Other medications	1,178.10 (300.60-3,098.88)
Total	52,700.72 (46,085.61-60,746.63)

The median annual direct medical cost was studied with reference to the status of different genetic polymorphisms of the studied gene of interest to the participants. Table 4 shows the total direct healthcare costs incurred based on the status of CYP3A5*3 genetic polymorphism. Results showed that out of the KTRs carrying the CYP3A5*1 wildtype allele (n = 28), 22 (78.6%) reported graft survival without transplant-related complications, while 38 (76.0%) among the 50 CYP3A5*3 variant alleles had graft survival without acute graft rejection and/or ATN. KTRs with CYP3A5*1 wildtype allele had a total annual median cost incurred of MYR 47,636.03 (IQR: 44,016.35-54,885.95), while a total median annual cost of MYR 59,292.03 (IQR: 50,414.70-62,486.79) was incurred among patients with CYP3A5*3 variant allele (p < 0.001). The ICER for achieving graft survival without acute graft rejection and/or ATN for KTRs with CYP3A5*3 variant allele compared to CYP3A5*1 wildtype allele was MYR 448,307.70.

**Table 4:** Median annual direct costs for outpatient, laboratory tests, hospitalization, biopsies, ISA, and other medications based on CYP3A5*3 polymorphism of the study population (n = 39) Note: cost in the year 2021 ISA, immunosuppressive agents; IQR, interquartile range; MYR, Malaysian Ringgit

Cost component	All patients (MYR), median (IQR)	CYP3A5*1 allele (wildtype) (MYR), median (IQR)	CYP3A5*3 allele (variant) (MYR), median (IQR)
Outpatient visit	6,360.00 (6,000.00-6,360.00)	6,360.00 (5,280.00-6,360.00)	6,360.00 (5,280.00-6,360.00)
Laboratory tests	21,349.00 (21,349.00-21,429.00)	21,349.00 (21,349.00-21,429.00)	21,349.00 (21,349.00-21,429.00)
Hospitalization	4,344.00 (780.00-6,907.80)	1,952.60 (260.00-3,9174.80)	4,912.40 (260.00-39,174.80)
Biopsies	500.00 (500.00-500.00)	500.00 (500.00-500.00)	500.00 (500.00-500.00)
ISA	19,432.66 (14,366.70-26,339.83)	16,962.71 (10,887.67-42,822.29)	26,339.83 (12,422.46-44,328.56)
Other medication	1,178.10 (300.60-3,098.88)	809.10 (32.40-19,850.85)	1,543.34 (32.40-19,850.85)
Total	52,700.72 (46,085.61-60,746.63)	48,281.61 (43,733.46-57,759.70)	60,257.67 (51,414.86-63,702.23)

**Figure 1:**
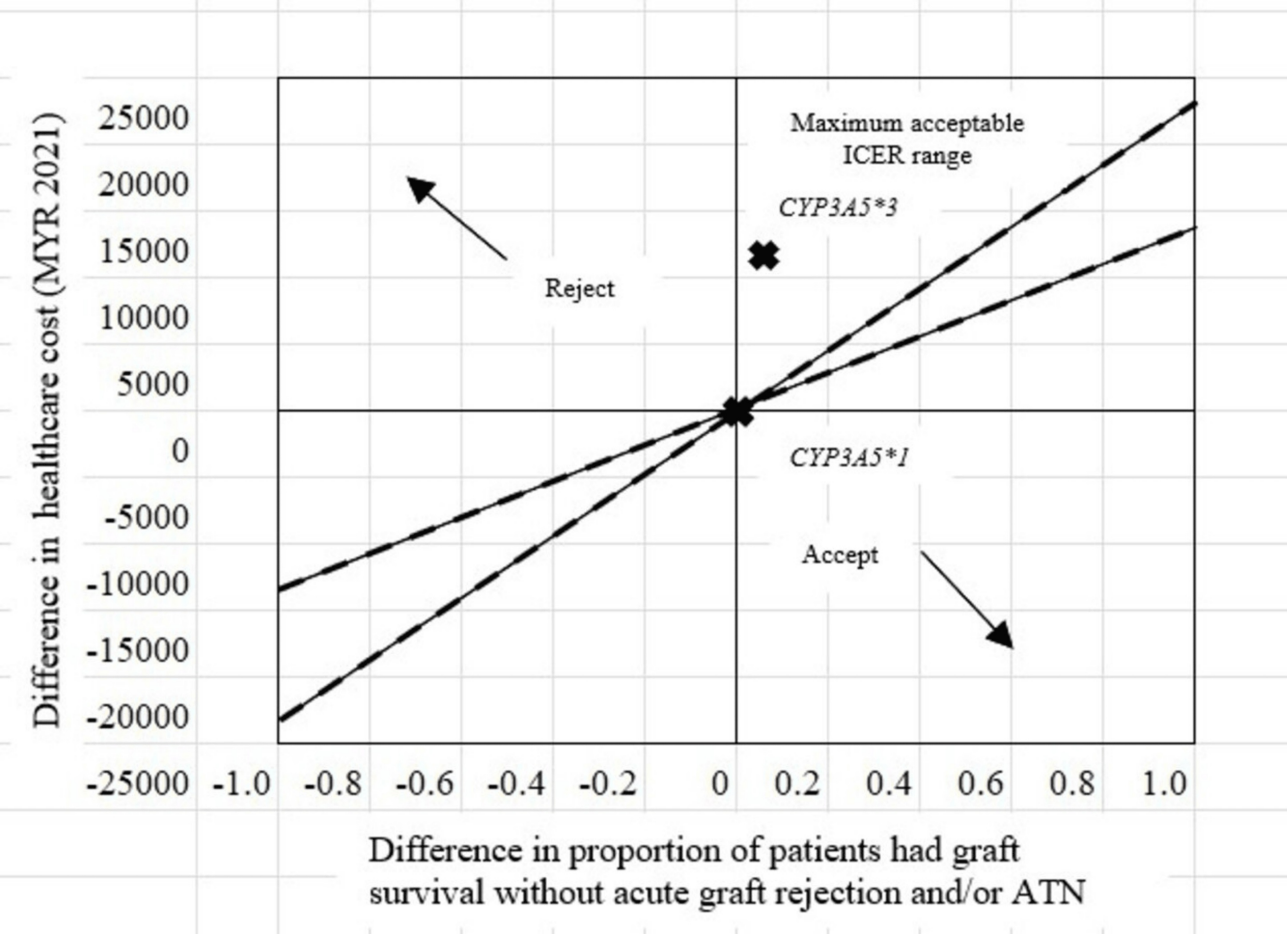
The location of the ICER (----) for graft survival with acute graft rejection and/or ATN among patients with CYP3A5*3 polymorphism in the cost-effectiveness plane

The incremental cost and the incremental effect were positive; thus, the ICER was found in the northeast quadrant of the cost-effectiveness plane (Figure 1). However, the ICER was located to the left of the maximum acceptable ICER range, which indicated that the management was able to achieve graft survival without acute graft rejection and/or ATN for patients with CYP3A5*3 variant allele compared to CYP3A5*1 wildtype allele, but incurred an additional cost of MYR 44,8 307.70, rendering this approach not cost-effective in achieving graft survival without acute graft rejection and/or ATN in CYP3A5*3 compared to CYP3A5*1 polymorphism.

Table 5 shows the total direct healthcare costs incurred based on the status of *ABCC2 -24C>T* genetic polymorphism. For *ABCC2 -24C>T* genetic polymorphism, it was found that KTRs with *ABCC2 -24C>T C* wildtype allele (n = 46, 74.2%) and *ABCC2 -24C>T T* variant allele (n = 14, 87.5%) reported graft survival without acute graft rejection and/or ATN. The median medical treatment costs incurred for *ABCC2 -24C>T* wildtype and variant carrier were MYR 50,434.74 (IQR: 47,003.67-60,056.35) and MYR 48,111.66 (IQR: 43,969.40-61,485.53), respectively (p = 0.552). The ICER for graft survival without acute graft rejection and/or ATN for KTRs with *ABCC2 -24C>T C* wildtype allele compared to the *ABCC2 -24C>T T* variant allele was (-) MYR 17,466.77.

**Table 5:** Median annual direct costs for outpatient, laboratory tests, hospitalization, biopsies, ISA, and other medications based on ABCC2 -24C>T polymorphism of the study population (n = 39) Note: cost in the year 2021 ISA, immunosuppressive agents; IQR, interquartile range; MYR, Malaysian Ringgit

Cost component	All patients (MYR), median (IQR)	*ABCC2 -24C>T C* allele (wildtype) (MYR), median (IQR)	*ABCC2 -24C>T T* allele (variant) (MYR), median (IQR)
Outpatient visit	6,360.00 (6,000.00-6,360.00)	6,360.00 (5,280.00-6,360.00)	6,360.00 (5,280.00-6,360.00)
Laboratory tests	21,349.00 (21,349.00-21,429.00)	21,349.00 (21,349.00-21,429.00)	21,349.00 (21,349.00-21,429.00)
Hospitalization	4,344.00 (780.00-6,907.80)	4,344.00 (260-39,174.80)	3,717.74 (260.00-11,218.47)
Biopsies	500.00 (500.00-500.00)	500.00 (500.00-500.00)	500.00 (500.00-500.00)
ISA	19,432.66 (14,366.70-26,339.83)	20,011.24 (10,887.67-44,328.56)	17,961.88 (11,741.24-44,050.50)
Other medication	1,178.10 (300.60-3,098.88)	1,516.50 (32.40-19,850.85)	749.70 (34.20-19,850.85)
Total	52,700.72 (46,085.61-60,746.63)	52,797.73 (47,309.69-60,530.89)	48,111.66 (43,310.49-62,407.39)

**Figure 2:**
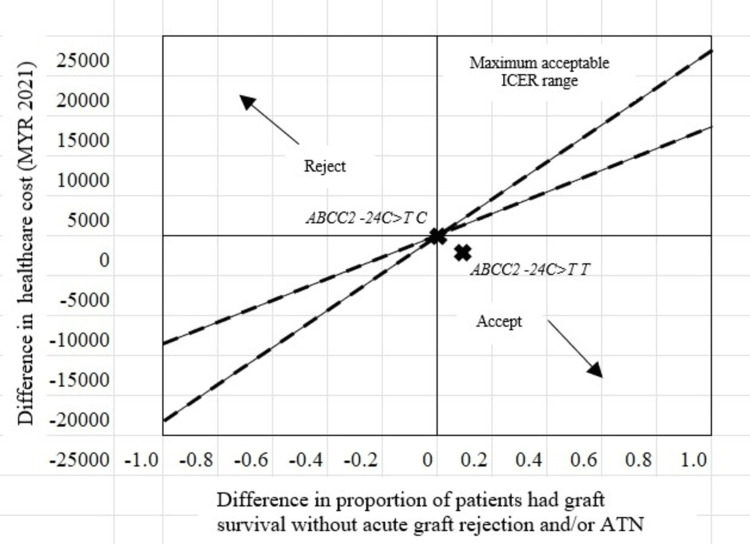
The location of the ICER (---) for graft survival with acute graft rejection and/or ATN among patients with ABCC2 -24C>T polymorphism in the cost-effectiveness plane

The incremental cost was negative, indicating that the cost of achieving graft survival without acute graft rejection and/or ATN among patients with a variant allele was less than the wildtype allele carrier *ABCC2 -24C>T*. However, the incremental effect was positive, indicating that it was more effective in managing patients with the *ABCC2 -24C>T T* variant allele compared to the *ABCC2 -24C>T C* wildtype allele. The ICER for achieving graft survival without acute graft rejection and/or ATN was at the southeast of the quadrant of the cost-effectiveness plane, reflecting that the current approach was more cost-effective among the *ABCC2 -24C>T T* variant allele carriers compared to the *ABCC2 -24C>T C* wildtype allele carriers (Figure 2).

Table 6 shows the total direct healthcare costs incurred based on the status of the *ABCC2 3972C>T* genetic polymorphism. It was observed that among KTRs, those carrying *ABCC2 3972C>T C* allele (n = 43, 76.8%) and *ABCC2 3972C>T T* allele (n = 17, 77.3%) reported graft survival without acute graft rejection and/or ATN, with a median medical treatment cost of MYR 50,434.74 (IQR: 46,085.61-59,292.03) and MYR 48,281.61 (IQR: 44,763.73-62,891.94), respectively (p = 0.961). The ICER for achieving graft survival without acute graft rejection and/or ATN for patients with *ABCC2 3972C>T T* variant allele compared to its wildtype allele was (-) MYR 430,626.00.

**Table 6:** Median annual direct costs for outpatient, laboratory tests, hospitalization, biopsies, ISA, and other medications based on ABCC2 3972C>T polymorphism of the study population (n = 39) Note: cost in the year 2021 ISA, immunosuppressive agents; IQR, interquartile range; MYR, Malaysian Ringgit

Cost component	All patients (MYR), median (IQR)	*ABCC2 3972C>T C* allele (wildtype) (MYR), median (IQR)	*ABCC2 3972C>T T* allele (variant) (MYR), median (IQR)
Outpatient visit	6,360.00 (6,000.00-6,360.00)	6,360.00 (5,280.00-6,360.00)	6,360.00 (5,280.00-6,360.00)
Laboratory tests	21,349.00 (21,349.00-21,429.00)	21,349.00 (21,349.00-21,429.00)	21,349.00 (21,349.00-21,429.00)
Hospitalization	4,344.00 (780.00-6,907.80)	4,344.00 (260.00-39,174.80)	3,168.20 (260.00-39,174.80)
Biopsies	500.00 (500.00-500.00)	500.00 (500.00-500.00)	500.00 (500.00-500.00)
ISA	19,432.66 (14,366.70-26,339.83)	19,500.23 (10,887.67-44,328.56)	19,069.79 (11,741.24-44,050.50)
Other medication	1,178.10 (300.60-3,098.88)	1,043.33 (32.40-19,850.85)	1,682.10 (34.20-19,850.85)
Total	52,700.72 (46,085.61-60,746.63)	52,797.73 (46,391.63-60,358.32)	51,843.48 (44,851.82-63,146.29)

**Figure 3:**
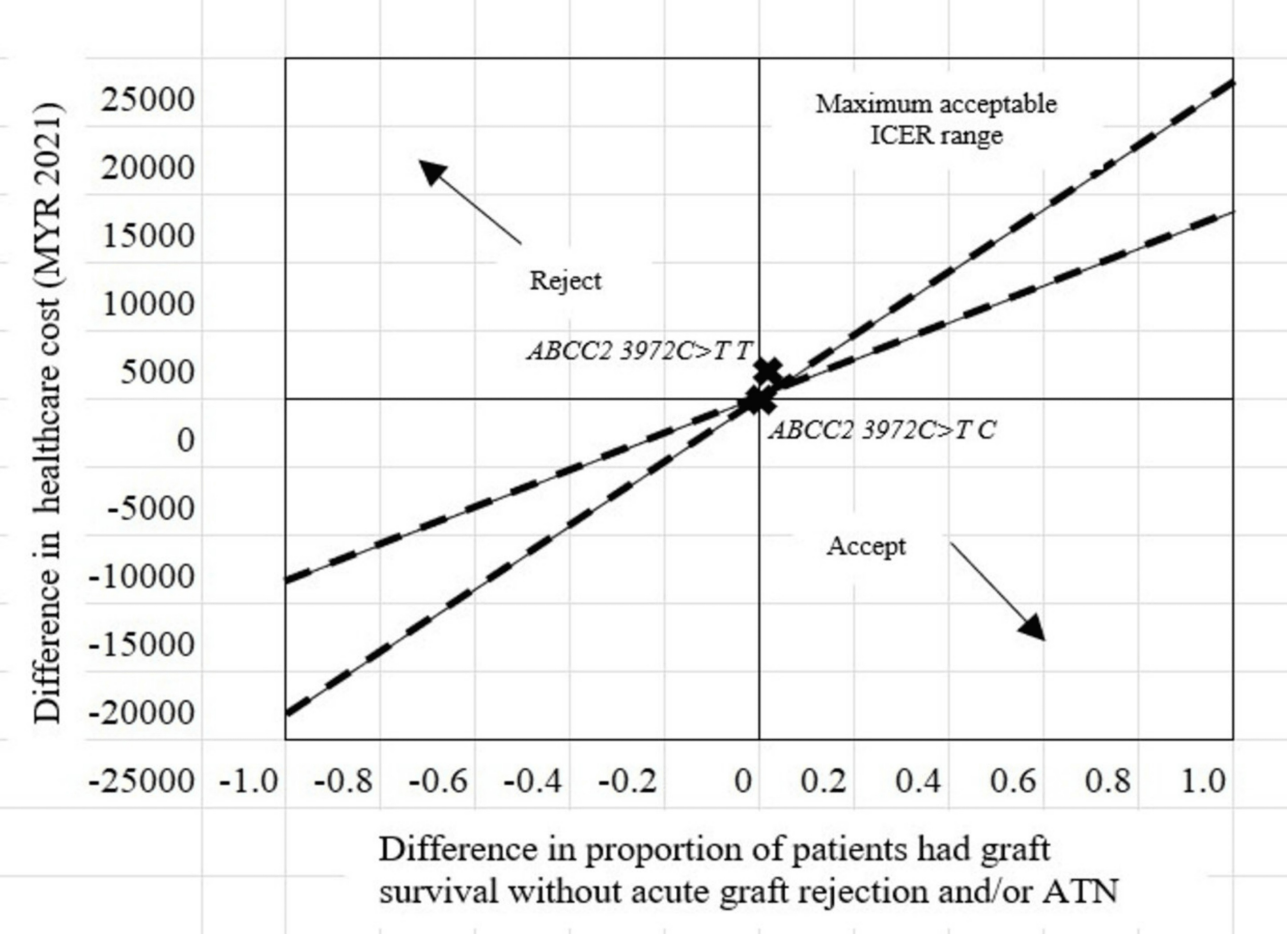
The location of the ICER (---) for graft survival with acute graft rejection and/or ATN among patients with ABCC2 3972C>T polymorphism in the cost-effectiveness plane

The cost incurred for achieving graft survival without acute graft rejection and/or ATN was demonstrated to be less among *ABCC2 3972C>T T* variant carriers compared to *ABCC2 3972C>T C* wildtype allele carriers. This demonstrated that it was more effective in achieving graft survival without acute graft rejection and/or ATN among patients carrying the variant allele than the wildtype allele of *ABCC2 3972C>T* gene. The ICER was located at the northeast quadrant of the cost-effectiveness plane and within the maximum acceptable ICER range (Figure 3).

## Discussion

This study investigated the financial impact of genetic variations on kidney transplantation. It estimated the overall cost of managing kidney transplants and identified a significant link between genetic polymorphisms in CYP3A5*3, *ABCC2 -24C>T*, and *ABCC2 3972C>T* and the cost of achieving successful graft survival without rejection or ATN. Laboratory tests emerged as the biggest cost driver among analyzed categories, followed by immunosuppressive medications. This reinforces existing knowledge about medication costs being a major factor [2]. However, our study identifies laboratory tests as another significant contributor. Standard hospital protocols mandate frequent lab tests post-transplant. These tests occur as often as three times weekly for the first six weeks, transitioning to once every three weeks after nine months. This doesn’t account for additional testing due to complications or hospitalizations. Pharmacogenetic testing before treatment could be a cost-saving strategy. Research suggests it can predict both treatment response and ADRs [6]. For example, Thervet et al. recommend a lower initial tacrolimus dose for patients with the CYP3A5*3/*3 genotype compared to normal *CYP3A5* expressers [7]. This approach allows a higher proportion of patients to reach the targeted tacrolimus level faster, potentially reducing costs associated with excess medication and preventing ADRs.

This study explored the cost-effectiveness of using genetic testing to tailor immunosuppressive therapy for kidney transplant patients. The results, analyzed using the ICER framework, positioned the intervention in the favorable northeast quadrant of the cost-effectiveness plane. It was demonstrated that while genetic testing for the CYP3A5*3 polymorphism might incur higher initial costs compared to a standard approach [13,28], it remains cost-effective in achieving graft survival without complications like rejection or ATN [9]. Several factors contribute to the potential cost increase. First, there’s the initial cost of the genetic test itself. Additionally, patients with the CYP3A5*3 polymorphism may require more frequent monitoring of blood levels for the immunosuppressant tacrolimus [29]. This close monitoring often necessitates dosage adjustments to maintain optimal drug concentration, potentially increasing short-term healthcare costs. However, this personalized approach through genetic testing offers significant long-term benefits. Ensuring the drug’s effectiveness while minimizing side effects and complications leads to better patient outcomes. This translates to potentially lower long-term healthcare needs associated with transplant rejection or medication toxicity.

The cost-effectiveness of pharmacogenetic testing for KTRs focused on analyzing the *ABCC2 -24C>T* polymorphism and its impact on achieving graft survival without acute graft rejection and/or ATN. The resulting ICER positioned this polymorphism within the southeast quadrant of the cost-effectiveness plane, suggesting that managing KTRs with the *ABCC2 -24C>T* variant allele (T) was more cost-effective compared to those with the wildtype allele (C) [13,28]. However, data also revealed a concerning trend in which a higher incidence of acute graft rejection and/or ATN was observed among KTRs harboring the wildtype allele C. While this observation suggests the potential cost-effectiveness of the T allele for graft survival, it necessitates further investigation to elucidate the underlying cause of the increased complication rates. Pending a more comprehensive understanding of this phenomenon, *ABCC2 -24C>T* genotyping in KTRs may still hold significant value. Identifying patients with the T variant allele could empower clinicians to implement more specific and timelier pharmacotherapeutic interventions, proactively preventing transplant-related complications. This underscores the need for further exploration into the application of *ABCC2 -24C>T* genotyping, particularly among KTRs prescribed tacrolimus-based immunosuppressant regimens.

When delving into the cost-effectiveness of pharmacogenetic testing for KTRs and assessing the impact of the *ABCC2 3972C>T* polymorphism on achieving graft survival without acute graft rejection and/or ATN, our analysis revealed ICER for this specific polymorphism positioned it within the northeast quadrant of the cost-effectiveness plane, hinting at a potential advantage for the presence of the *ABCC2 3972C>T* variant allele (T) over the wildtype allele (C) [13,28]. However, a more profound examination uncovers a nuanced relationship between cost and clinical outcomes. While the T allele may suggest a more cost-effective pathway to graft survival, our data hints at a correlation with a lower incidence of transplant-related complications, particularly acute graft rejection and/or ATN. This seemingly contradictory observation demands further exploration, suggesting that while the T allele might incur higher healthcare costs, it could also lead to improved patient outcomes by mitigating the need for additional interventions. Studies by Clayton et al. underscore the potential benefits of personalized medicine in enhancing graft and patient survival rates that demonstrate the significance of conducting *ABCC2 3972C>T* genotyping before kidney transplantation [30]. By identifying patients with the T variant allele, clinicians gain the ability to tailor immunosuppressive therapy regimens more precisely [6]. This personalized approach, guided by a patient’s unique genetic profile, not only optimizes cost-effectiveness but also holds promise for enhancing patient outcomes by potentially reducing the incidence of transplant-related complications and thereby improving graft and patient survival rates. Further research is warranted to fully unravel the underlying mechanisms and refine tailored immunosuppressive regimens based on a patient’s *ABCC2 3972C>T* genotype.

Among the limitations of the study is the sole focus on direct cost. Indirect costs, such as lost income and productivity losses due to work inefficiency, were not evaluated. These indirect costs can be substantial and may significantly impact the overall economic burden of a disease. Additionally, the generalizability of the findings may be limited due to the study population being restricted to patients in public tertiary care hospitals. Patients in private healthcare settings may experience different cost structures, and further research is needed to determine if the observed cost trends hold true in this population. 

## Conclusions

To the best of our knowledge, this is the first study demonstrating carriers of the CYP3A5*1 wildtype allele, the *ABCC2 -24C>T T* variant allele, and the *ABCC2 3972C>T T* variant allele were more cost-effective in lowering the risk of ATN and graft rejection among KTRs. Our findings underscore the promising potential of novel findings of CYP3A5*3, *ABCC2 -24C>T*, and *ABCC2 3972C>T* genotyping in identifying KTRs who may incur escalated healthcare costs. Equipped with this genetic insight, healthcare practitioners can tailor immunosuppressive treatment plans, potentially mitigating the necessity for additional interventions and alleviating the financial burden on healthcare systems involved in KTR care. This personalized approach not only holds promise for optimizing patient outcomes but also contributes to the sustainability of healthcare resources, ensuring efficient allocation and enhanced quality of care for KTRs.
